# 

**Published:** 1874-08

**Authors:** 


THREE DOLLARS A YEAR. SPECIMEN COPIES, 30 CENTS.

VOL. XXXI. August, 1874. NUMBER 8.

THE

### Chicago Medical Journal.

J. ADAMS ALLEN, M.D., LL.D., WALTER HAY, M.D.,
EDITORS.

TWELVE TIMES A YEAR.

CHICAGO:
W. B. KEEN, COOKE & CO., PUBLISHERS,
NOS. 113 AND 115 STATE STREET.

